# Effects of organic and inorganic selenium on selenium bioavailability, growth performance, antioxidant status and meat quality of a local beef cattle in China

**DOI:** 10.3389/fvets.2023.1171751

**Published:** 2023-04-27

**Authors:** Qi Huang, Shuiping Wang, Xin Yang, Xuefeng Han, Yong Liu, Nazir Ahmad Khan, Zhiliang Tan

**Affiliations:** ^1^CAS Key Laboratory for Agro-ecological Processes in Subtropical Region, National Engineering Laboratory for Pollution Control and Waste Utilization in Livestock and Poultry Production, Hunan Provincial Key Laboratory of Animal Nutritional Physiology and Metabolic Process, Institute of Subtropical Agriculture, Chinese Academy of Sciences, Changsha, Hunan, China; ^2^Chongqing Key Laboratory of Herbivore Science, College of Animal Science and Technology, Southwest University, Chongqing, China

**Keywords:** antioxidant capacity, meat quality, selenium sources, selenium bioavailability, selenium tissue deposition

## Abstract

Selenium (Se) is an essential nutrient with multiple health benefits to humans and animals. Cattle generally require dietary Se supplementation to meet their daily requirements. The two main forms of dietary Se in cattle are organic Se and inorganic Se. Data comparing the health and productivity effects of organic Se and inorganic Se on cattle are still insufficient, and it is necessary to conduct more research to evaluate the bioavailability, nutritional value, deposition, and body functions of Se sources in different breeds and physiological stages of cattle raised in areas with different Se levels. The objectives of this study were to determine the effects of organic and inorganic sources of Se on plasma biochemical indices, Se bioavailability, deposition in body tissues and organs, growth performance, antioxidant capacity and meat quality of beef cattle raised in Se-deficient areas. Fifteen Chinese Xiangzhong Black beef cattle with an average weight of 254.5 ± 8.85 kg were assigned to three dietary groups. The three groups were fed the same basal ration and supplemented with either an inorganic [sodium selenite (SS)] or organic [selenomethionine (SM) or Se-enriched yeast (SY)] source of Se (0.1 mg/kg dry matter) for 60 days. At the end of the experiment, three cattle from each group were randomly selected and slaughtered, and samples were collected from tissues and organs for analysis. The results revealed that growth performance, slaughter performance, Se content of tissues and organs, meat quality characteristics including chemical composition, pH_45min_, pH_24h_, drip loss, and cooking losses did not differ (*p* > 0.05) due to supplementation of the different organic and inorganic sources of Se. SM and SY were more effective in increasing (*p* < 0.05) immunoglobulin M (IgM) concentrations in the blood and reducing (*p* < 0.05) malondialdehyde (MDA) content in the longissimus dorsi than SS. In conclusion, organic Se is more effective than inorganic Se in improving the immune and antioxidant capacity of Chinese Xiangzhong Black beef cattle.

## 1. Introduction

Selenium (Se) is a nutritionally essential trace element for mammals. Se functions as a constituent of over 30 selenoproteins and plays an important role in antioxidant defense and maintenance of redox homeostasis (1). Dietary Se supplementation in cattle has been shown to improve rumen fermentation and nutrient absorption and utilization (2–4). Moreover, Se supplementation promotes growth (5), reproduction (6), milk yield and quality in cattle (7). In addition to meeting animal requirements, Se supplementation in feed can also increase Se content of animal products, which is considered an important intervention in minimizing the risk of Se deficiency in humans (8, 9). China is one of the 40 countries designated low Se or Se deficient according to the World Health Organization (WHO), and Se-deficient areas account for 72% of the country’s total area, which affects over 70 million people who face potential adverse health impacts due to Se deficiency (10, 11). Se deficiency (Se intake <10 μg/day) is considered to be responsible for the widespread prevalence of cardiomyopathy, which is also linked to Keshan disease and Kashin-Beck disease (12, 13). The Se level in the human population depends on long-term daily intake. Beef is an important daily dietary Se source for human beings. A safe and effective increase in Se content in beef is becoming a challenging concern that requires the adequate understanding of the absorption, metabolism, and deposition of Se in cattle.

Recent research has shown that organic Se sources, such as selenomethionine (SM) and Se-enriched yeast (SY), have higher bioavailability, smaller environmental footprint and lower toxic effects than conventional inorganic sources (14, 15). Due to the similarity between SM and methionine (Met), SM is more easily absorbed by the gut and incorporated into tissue protein, thus forming an endogenous Se reserve that can be utilized during periods of stress for additional synthesis of selenoproteins (16). It is generally believed that the absorption and utilization of inorganic Se in ruminants is lower than that in monogastric animals, which may be due to the metabolism and utilization of inorganic Se by the rumen environment (17). Studies have shown that rumen microorganisms can better absorb and protect organic Se, and SS is more easily converted into an insoluble inorganic form by rumen microorganisms (18). Some studies have reported that organic Se is more suitable than inorganic Se as a dietary supplement for animals, but the results from these studies have been inconsistent and sometimes contradictory (19, 20). Moreover, the underlying mechanisms for bioavailability, biological activity, and tissues deposition of Se sources are not fully understood.

To our knowledge, studies on the effect of inorganic and organic Se sources on the performance of Chinese local beef cattle are scarce. To better understand Se physiological functions and accumulation in cattle, it is necessary to conduct more research in different breeds and physiological stages or statuses of cattle to evaluate the bioavailability and deposition of Se sources. Therefore, the present study was designed to evaluate the effects of organic and inorganic sources of Se on plasma biochemical indices, Se bioavailability, Se tissues deposition, growth performance, antioxidant capacity and meat quality of Chinese indigenous beef cattle.

## 2. Materials and methods

All procedures for the animal trial were in accordance with the guidelines for animal care and use and approved (No. ISA000201) by the animal care committee of the Institute of Subtropical Agriculture, Chinese Academy of Sciences, Changsha, China.

### 2.1. Experimental animals, feeding and management of beef cattle

Fifteen healthy and similar age (10 months old) Chinese indigenous beef cattle (Xiangzhong Black) steers with an average body weight (BW) of 254.5 ± 8.85 kg were selected for the experimental trial. The experimental animals were assigned to three dietary groups. The three dietary groups received the same basal diet, formulated according to the Feeding Standard for Beef Cattle (NY/T 815–2004) of the National Standards of the People’s Republic of China (Table 1), and supplemented with 0.1 mg Se/kg DM, either from an inorganic [sodium selenite (SS)] or organic [selenomethionine (SM) or Se-enriched yeast (SY)] source for 60 days. The SS was provided by Sinopharm Chemical Reagent Co., Ltd. (Shanghai, China), and SM and SY were provided by Alltech (Kentucky, United States). Known quantity (corresponding to *ca.* 0.1 mg Se/kg DM of the diet) of each Se source was mixed and diluted sequentially with premix and concentrate. Concentrate was fed daily to the animals at 1% BW, and rice straw was fed *ad libitum*. Concentrate was always completely eaten daily by every cattle during the whole experiment. The experimental period was 70 days, consisting of a pretrial adaptation period of 10 days and a trial period of 60 days. The experimental animals were individually housed and fed twice daily in tie stalls, with free access to clean water throughout the experiment.

**Table 1:** Ingredients and chemical compositions of the basal diet.

Ingredients	(g/kg of DM)	Nutrition levels^b^	(g/kg of DM)
Corn	690.0	DM	874.0
Soybean meal	245.0	NEg (MJ/kg)	6.35
NaCl	10.0	CP	162.0
NaHCO_3_	10.0	EE	28.8
Limestone	10.0	NDF	87.8
CaHPO_4_	15.0	ADF	41.5
Premixes^a^	20.0	Ca	8.10
Total (g)	1000.0	P	6.10

a Premixes contained (per kg): Zn, 2 g; Cu, 500 mg; Fe, 2.5 g; Mn, 1.5 g; Co, 40 mg; I, 30 mg; vitamin A, 200 KIU; vitamin D, 110 KIU; vitamin E, 5 KIU. Se was provided to basal diet in three forms, including sodium selenite (Na_2_SeO_3_), seleno-methionine (SM, C5H11NO2Se), and selenium yeast.

b DM, Dry matter; NEg, Net energy for gain; CP, Crude protein; EE, Ether extract; NDF, Neutral detergent fibre; ADF, Acid detergent fibre; Ca, Calcium; P, Phosphorus.

### 2.2. Sampling and data collection

Feed samples were collected weekly and subsequently oven-dried at 65°C for 48 h. The dried feed samples were ground through a 1-mm screen using a Wiley mill (FW-100, Yongguangmin Ltd., Beijing, China) and then analysed for the contents of DM (method 930.15), crude protein (CP; method 984.13), ash by ignition at 600°C for 2 h (method 942.05), ether extract (EE; method 920.39) by the Soxhlet extraction method with diethyl ether, and acid detergent fibre (ADF; method 973.18) using the standard methods of AOAC (2005) (21). The neutral detergent fibre (NDF) content was determined following the methods of Van Soest et al. (22). The values were corrected for residual starch and protein using heat-stable *α*-amylase and sodium sulfite during the analysis. Both NDF and ADF were expressed inclusive of residual ash. All measurements were performed in triplicate.

At the beginning and end of the experiment, each cattle were weighed for two consecutive days before morning feeding. Average daily gain (ADG) values were calculated from weight measurements. At the end of the 60-day feeding period, three animals from each dietary group were randomly selected and slaughtered after a 12 h fasting period. Hot carcass weight (HCW) of the slaughtered animals was measured immediately after evisceration. Carcass yield was expressed as a dressing percentage. After slaughtering, the heart, lungs, liver, kidney, spleen, and pancreas of each cattle were weighed. The organ weight index was calculated by dividing the organ weight of each animal by its body weight.


$$\mathrm{Dressing}\;\left(\%\right)=\frac{\mathrm{Hot}\;\mathrm{carcass weight}\;\left(\mathrm{kg}\right)}{\mathrm{Live body weigh}\;\left(\mathrm{kg}\right)}\times 100$$

Samples (*ca.* 200 g) from the longissimus dorsi (between the 9th and 11th ribs), semimembranosus, and semitendinosus were collected from the right side of each carcass for evaluation of meat quality characteristics and chemical composition. Meat quality was assessed by determining the changes in pH over time, drip loss, and cooking loss of the muscle samples. The pH values were measured 45 min (pH_45min_) and 24 h (pH_24h_) after slaughter using a pH meter (pH-STAR, Matthäus GmbH & Co. KG, Germany). Cooking losses were determined using the method described by Honikel (23). Briefly, meat samples (*ca.* 100 g, 50 mm thick) were cooked in a temperature-controlled water bath in thin-walled heat-resistant plastic bags to an internal temperature of 75°C, with the bag opening extending above the water surface. During this process, the internal temperature of the sample was measured with a digital thermometer. Samples were weighed before and after cooking. Cooking loss was calculated from the difference between the weight of the raw and cooked samples and expressed as a percentage of the initial weight. Drip losses were determined as weight loss during the suspension of a standardized sample (2 × 2 × 1 cm), sealed in a polyethylene bag, at 4°C after 48 h of storage. The muscle samples were also analyzsed for the contents of CP, EE, and ash according to the methods of AOAC (2005) (21). All measurements were performed in triplicate.

Blood samples were collected from the coccygeal vein of each cattle before the morning feeding on day 0, 20, 40, and 60 of the experiment. Each blood sample was placed in a heparinized vacuum tube (Aosaite, China). Plasma was collected after centrifugation of the blood samples at 3,000 × g for 15 min and immediately stored at −20°C for further biochemical analysis. Plasma samples were analysed for concentration of immunoglobulin M (IgM), alanine aminotransferase (ALT), aspartate transaminase (AST), alkaline phosphatase (ALP), creatine kinase (CK), ammonia (NH_3_), total protein (TP), and albumin (ALB) using the Mindray BS-300 automatic biochemical analyser (Shenzhen, China).

Tissue samples (*ca.* 50 g) from the liver, longissimus dorsi, semimembranosus, and semitendinosus were collected, quickly frozen with liquid nitrogen and stored at −80°C for further analysis of antioxidant status. The concentrations of glutathione (GSH) and malondialdehyde (MDA) and the activities of the antioxidant enzymes glutathione peroxidase (GSH-PX) and gamma-glutamyl transferase (GGT) were determined by spectrophotometric methods using commercially available kits (Jiancheng Bioengineering Institute, Nanjing, China).

The hair was taken from the dorsal area and thoroughly cleaned with acetone and distilled water, according to recommendations by Górski (24). The Se contents of plasma, hair, longissimus dorsi, semimembranosus, and semitendinosus were determined using an atomic fluorescence spectrometer (AFS-830, Titan Instruments, Beijing, China) according to the national standard method (GB5009.93–2017) of China (25).


$$X=\frac{\left(A-A_{0}\right)\times V}{M\times 1000}$$

In this formula: X: The content of Se in the sample (mg/kg or mg/L); A: The mass concentration of Se in the sample solution (μg/L); A_0_: The mass concentration of Se in the blank solution (μg/L); V: The total volume of digestive fluid of sample (mL); M: The quantity or volume of sample (g or mL).

### 2.3. Statistical analysis

All statistical analyses were performed by GraphPad Prism (version 8.0) and SPSS 26.0 (IBM SPSS Statistics 26.0). A general linear model was used to analyses the effect of Se sources on plasma biochemical parameters at multiple time points using SPSS 26.0 (IBM SPSS Statistics 26.0). Data on the effect of Se sources on growth performance, antioxidant capacity, carcass characteristics, meat quality and Se content of body tissues and organs were analysed using one-way ANOVA. The effect of the Se source was considered significant if statistical tests yielded a *p* < 0.05 for a particular parameter. For parameters with a significant effect of the Se sources, *post hoc* analysis was performed using Tukey–Kramer multiple comparisons test to analyses the statistical significance of pairwise differences among the means.

## 3. Results

### 3.1. Growth performance

**Figure 1:**
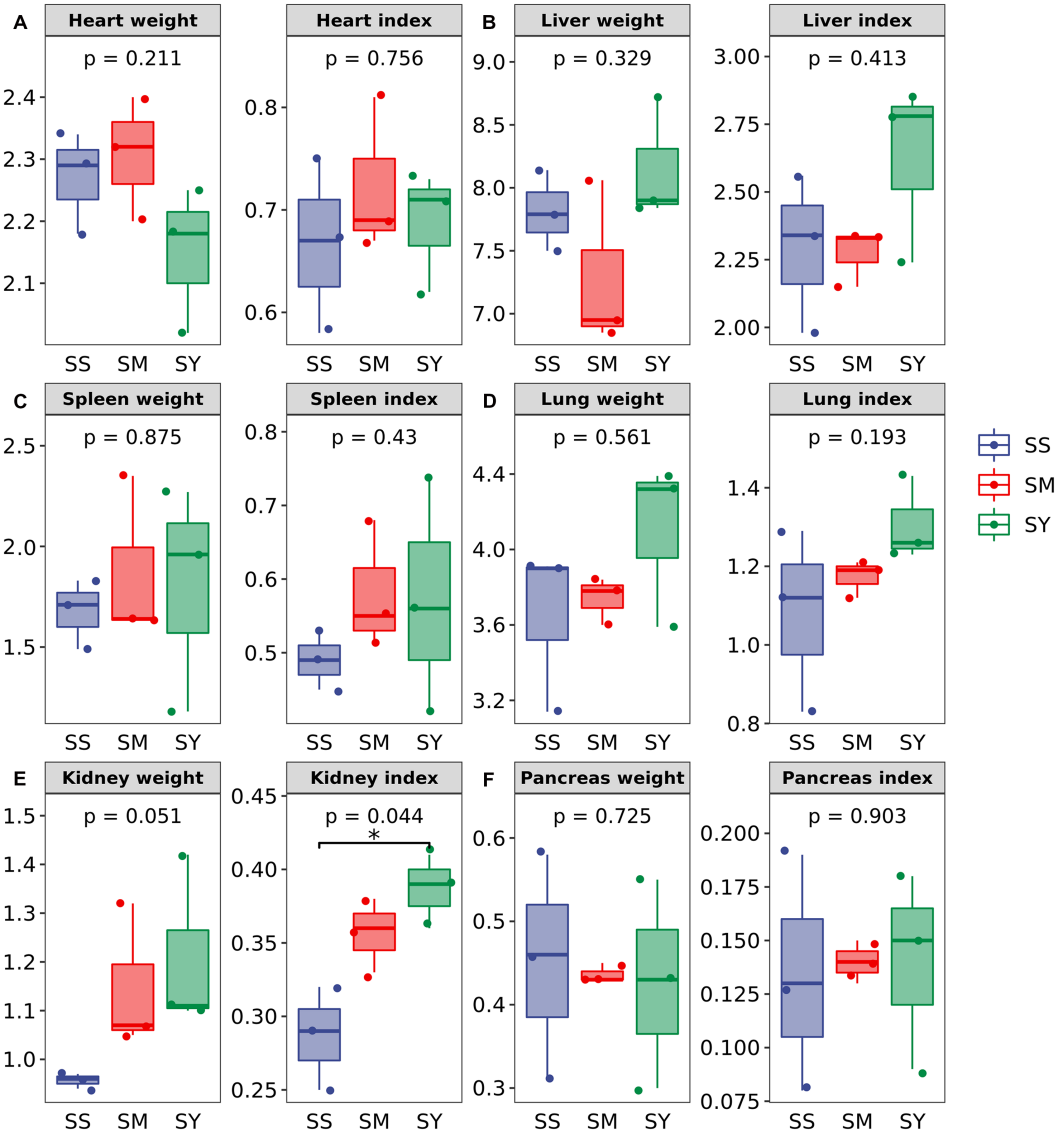
Effects of different Se sources on the organ weight and organ index of beef cattle (*n* = 3). **(A)** Heart; **(B)** Liver **(C)** Spleen; **(D)** Lung; **(E)** Kidney; **(F)** Pancreas. *p < 0.05; **p < 0.01. SS, diet supplemented with 0.1 mg Se/kg DM from sodium selenite; SM, diet supplemented 0.1 mg Se/kg DM from selenomethionine; SY, diet supplemented with 0.1 mg Se/kg DM from Se-enriched yeast.

The FBW, ADG, carcass weight and yield, organ weight and indices did not differ (*p* > 0.05) among Se sources (Figure 1; Table 2). However, the kidney weight index varied (*p* < 0.05) due to the Se sources, and the highest index was recorded for SY (Figure 1).

**Table 2:** Effects of different Se sources on the growth performance and carcass characteristics of beef cattle.

Item^b^	Se sources^a^			*p* value
	SS	SM	SY	
IBW kg	256.1 ± 17.45	254.2 ± 16.04	253.1 ± 16.09	0.992
FBW kg	318.8 ± 19.46	318.4 ± 16.30	313.2 ± 15.81	0.968
ADG kg/d	1.00 ± 0.04	1.02 ± 0.03	0.95 ± 0.07	0.649
Carcass weight kg	187.8 ± 11.30	168.8 ± 4.58	166.5 ± 9.02	0.248
Carcass yield %	54.7 ± 0.68	52.8 ± 0.86	53.2 ± 1.15	0.368

a SS, sodium selenite; SM, selenomethionine; SY, Se-enriched yeast.

b IBW, initial body weight; FBW, final body weight; ADG, average daily gain.

### 3.2. Meat quality

The results showed no differences (*p* > 0.05) in the contents of CP, EE, Ash, meat quality characteristics, including pH_45min_, pH_24h_, drip loss, and cooking losses among different Se sources (Table 3).

**Table 3:** Effects of different Se sources on the meat quality and composition of beef cattle.

Item	Se sources^a^			*p* value
	SS	SM	SY	
*Semitendinosus*				
pH_45min_	6.56 ± 0.11	6.39 ± 0.29	6.47 ± 0.03	0.802
pH_24h_	5.74 ± 0.05	5.88 ± 0.09	6.03 ± 0.05	0.073
Drip loss %	5.10 ± 0.19	4.14 ± 0.83	3.00 ± 0.91	0.205
Cooking loss %	55.2 ± 3.40	46.7 ± 0.71	52.2 ± 5.27	0.314
CP %	43.3 ± 1.39	44.1 ± 0.26	43.7 ± 0.71	0.816
Ash %	3.16 ± 0.09	3.57 ± 0.26	3.49 ± 0.18	0.370
EE %	9.68 ± 1.59	8.03 ± 0.32	7.90 ± 0.71	0.446
*Semimembranosus*				
pH_45min_	6.45 ± 0.12	6.78 ± 0.05	6.46 ± 0.15	0.151
pH_24h_	5.89 ± 0.19	6.02 ± 0.06	5.75 ± 0.03	0.359
Drip loss %	2.14 ± 0.54	3.77 ± 0.58	3.59 ± 1.34	0.426
Cooking loss %	50.9 ± 1.05	47.1 ± 1.31	49.4 ± 3.53	0.514
CP %	44.2 ± 0.92	44.0 ± 0.28	43.2 ± 0.83	0.646
Ash %	3.16 ± 0.09	3.41 ± 0.22	3.22 ± 0.04	0.487
EE %	9.26 ± 0.63	7.34 ± 1.37	9.94 ± 0.92	0.254
*Longissimus dorsi*				
pH_45min_	6.45 ± 0.11	6.64 ± 0.03	6.59 ± 0.37	0.830
pH_24h_	5.75 ± 0.13	5.88 ± 0.02	5.74 ± 0.02	0.449
Drip loss %	2.96 ± 0.89	3.95 ± 1.03	2.09 ± 0.07	0.318
Cooking loss %	53.5 ± 5.15	43.4 ± 0.66	50.8 ± 0.36	0.125
CP %	42.5 ± 1.44	42.0 ± 1.04	41.9 ± 1.27	0.944
Ash %	3.24 ± 0.05	3.35 ± 0.10	3.07 ± 0.14	0.249
EE %	10.2 ± 2.14	10.4 ± 1.65	10.3 ± 2.55	0.998

a SS, sodium selenite; SM, selenomethionine; SY, Se-enriched yeast.

### 3.3. Se concentration of plasma, muscles and organs

**Figure 2:**
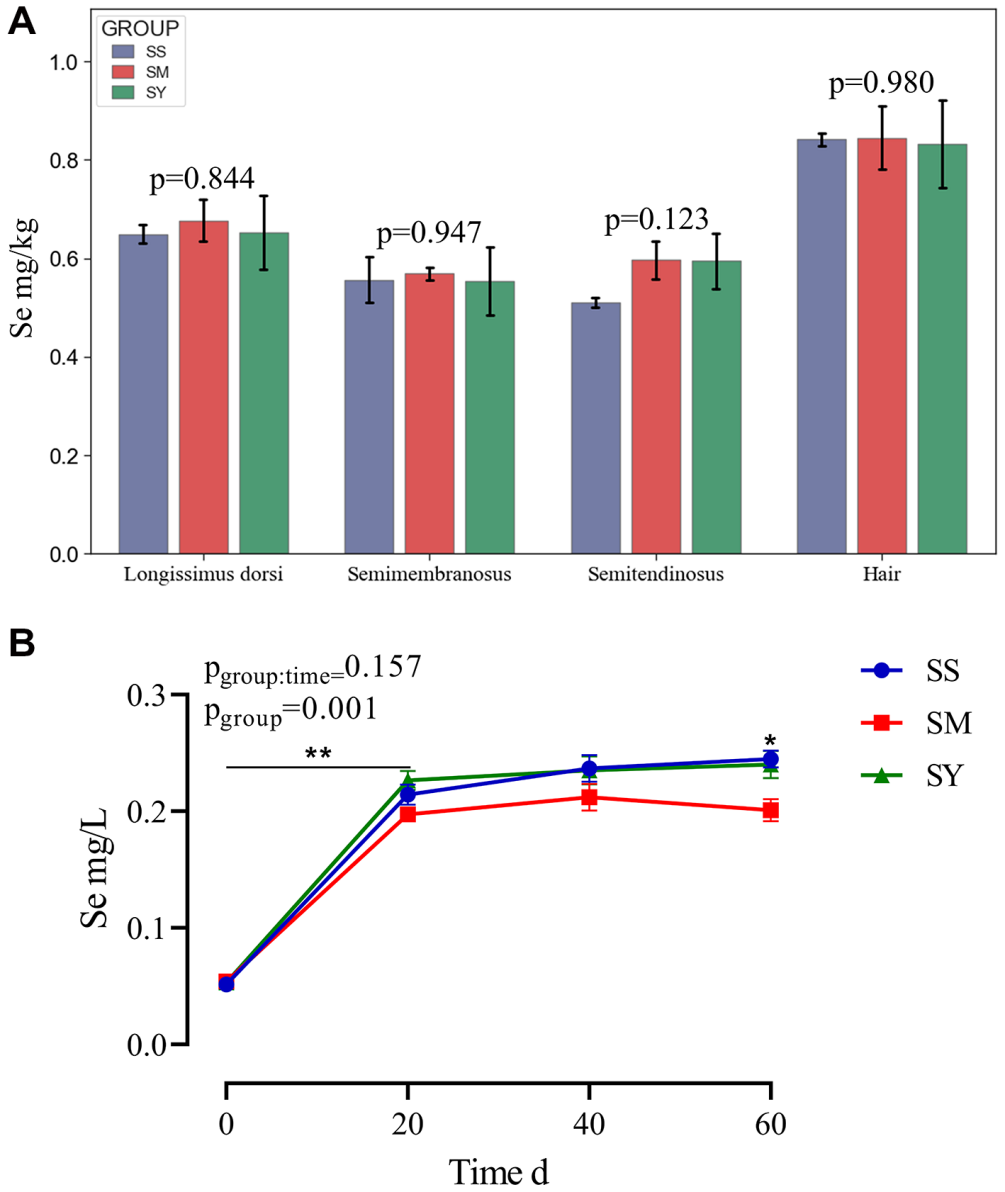
Se concentrations in muscle, hair and plasma of beef cattle over the 60 d feeding period. **(A)** The bar graphs (mean with SD; *n* = 3) showing the Se concentrations in muscle and hair on day 60; **(B)** The line chart (mean with SD; n=5) showing the plasma Se concentration on day 0, 20, 40 and 60. *p < 0.05, **p < 0.01. SS, diet supplemented 0.1 mg Se/kg DM from sodium selenite; SM, diet supplemented 0.1 mg Se/kg DM from; SY, diet supplemented 0.1 mg Se/kg DM from.

Irrespective of the sources, the plasma Se concentration was markedly higher (*p *< 0.01) in beef cattle supplemented with Se (0.1 mg/kg DM) on day 20, 40 and 60 than on day 0 (Figure 2B). Further comparisons revealed that the plasma Se concentration of the SM group was lower (*p* < 0.05) on day 60 than that of the SS and SY groups. However, at the end of the experiment, Se deposition in the semitendinosus, semimembranosus and longissimus dorsi muscles and hair did not differ (*p* > 0.05) among Se sources (Figure 2A).

### 3.4. Blood metabolites

**Figure 3:**
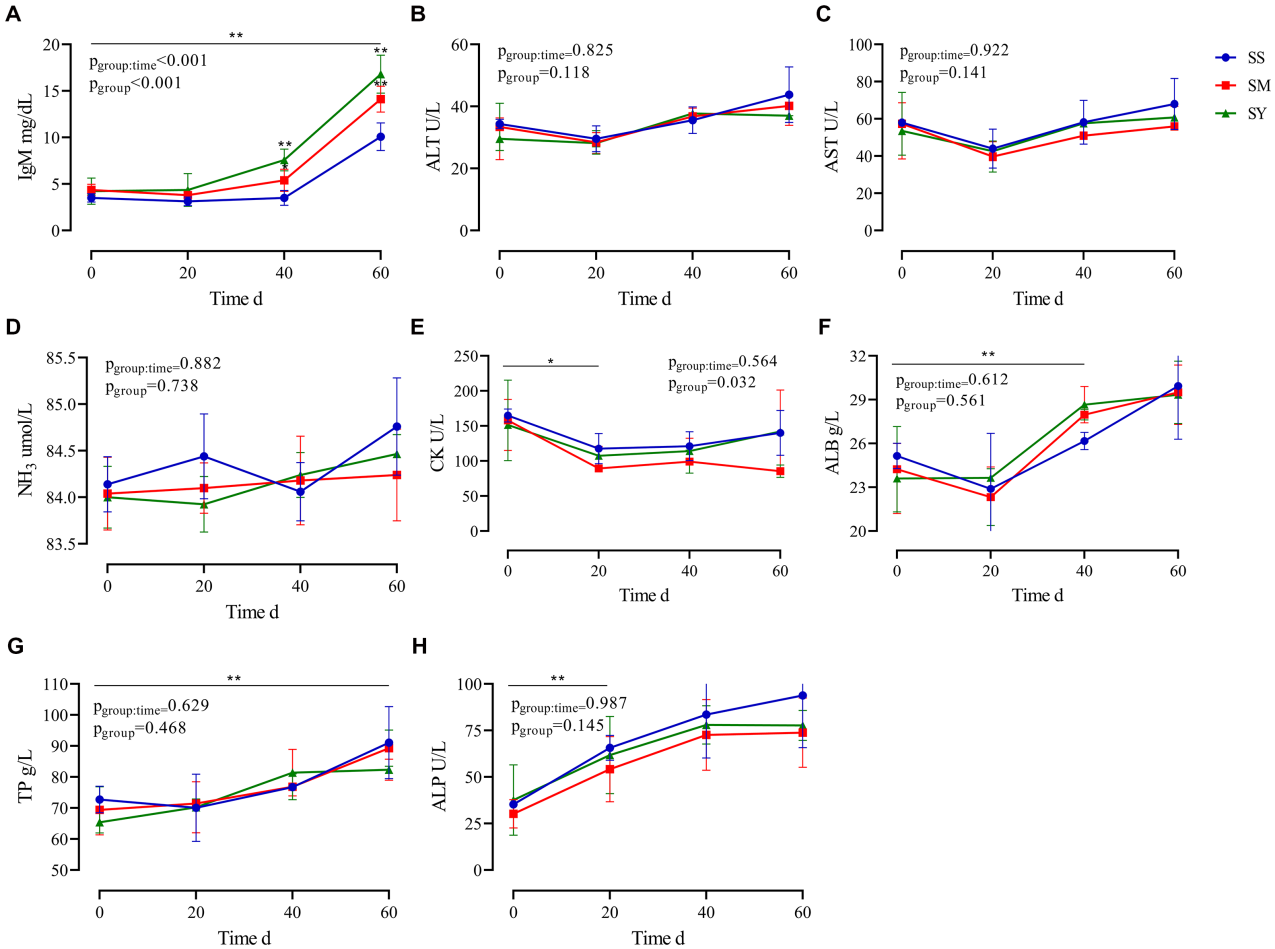
Effects of different Se sources on the plasma biochemical indices of beef cattle (*n* = 5) over the 60 d feeding period. **(A)** immunoglobulin M (IgM); **(B)** alanine aminotransferase (ALT); **(C)** aspartate transaminase (AST); **(D)** ammonia (NH_3_); **(E)** creatine kinase (CK); **(F)** albumin (ALB); **(G)** total protein (TP) and **(H)** alkaline phosphatase (ALP). *p < 0.05, **p < 0.01. SS, diet supplemented 0.1 mg Se/kg DM from sodium selenite; SM, diet supplemented 0.1 mg Se/kg DM from selenomethionine; SY, diet supplemented 0.1 mg Se/kg DM from Se-enriched yeast.

The results revealed that the plasma IgM concentration was higher (*p* < 0.01) in the SY and SM groups than in the SS group. The supplementation period affected (*p* < 0.01) the plasma IgM concentration, with higher (*p* < 0.01) IgM concentrations on day 60 in all groups. Plasma CK levels decreased (*p* < 0.05) after Se supplementation, however, there was no difference (*p* > 0.05) among the three sources of Se. Irrespective of the source, the plasma concentrations of ALB, TP, and ALP increased (*p* < 0.01) after 60 days of Se supplementation. However, the plasma concentrations of ALB, TP, and ALP on day 0, 20 and 60 were not different (*p* > 0.05) among Se sources. The plasma concentrations of ALT, AST, NH_3_, and GGT did not vary (*p* > 0.05) after Se supplementation (Figure 3).

### 3.5. Antioxidant capacity

**Figure 4:**
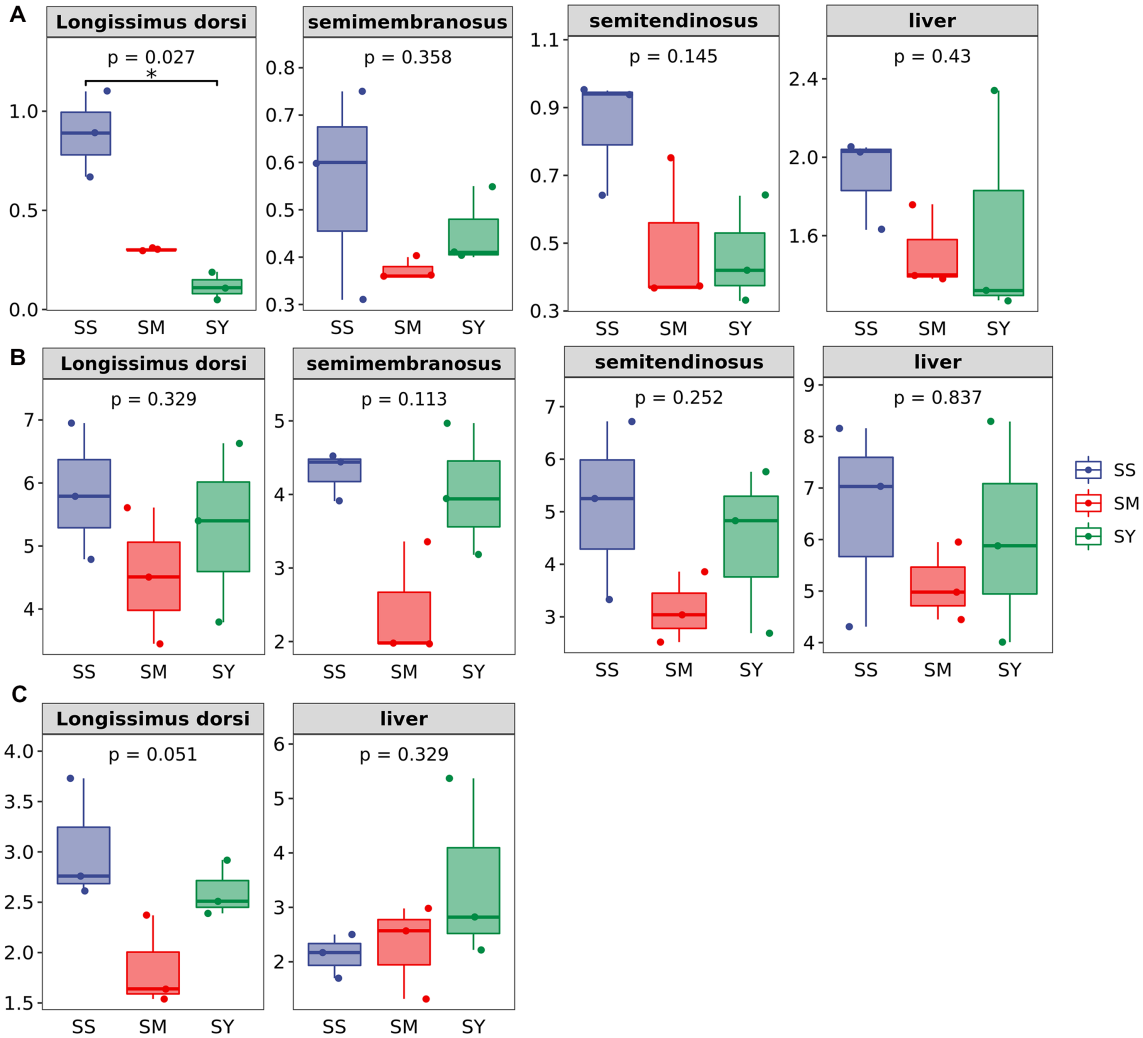
Effects of different Se sources on concentrations of MDA and GSH and GSH–Px activities in muscle and liver of beef cattle (*n* = 3). **(A)** malondialdehyde (MDA) concentrations in longissimus dorsi (LD); semimembranosus (SM), and semitendinosus (ST) and Liver; **(B)** antioxidant enzyme glutathione peroxidase (GSH-PX) activity of LD, SM, ST and Liver; **(C)** glutathione (GSH) concentrations of LD and Liver. *p < 0.05, **p<0.01. SS, diet supplemented 0.1 mg Se/kg DM from sodium selenite; SM, diet supplemented 0.1 mg Se/kg DM from selenomethionine; SY = with 0.1 mg Se/kg Se-enriched yeast.

**Figure 5:**
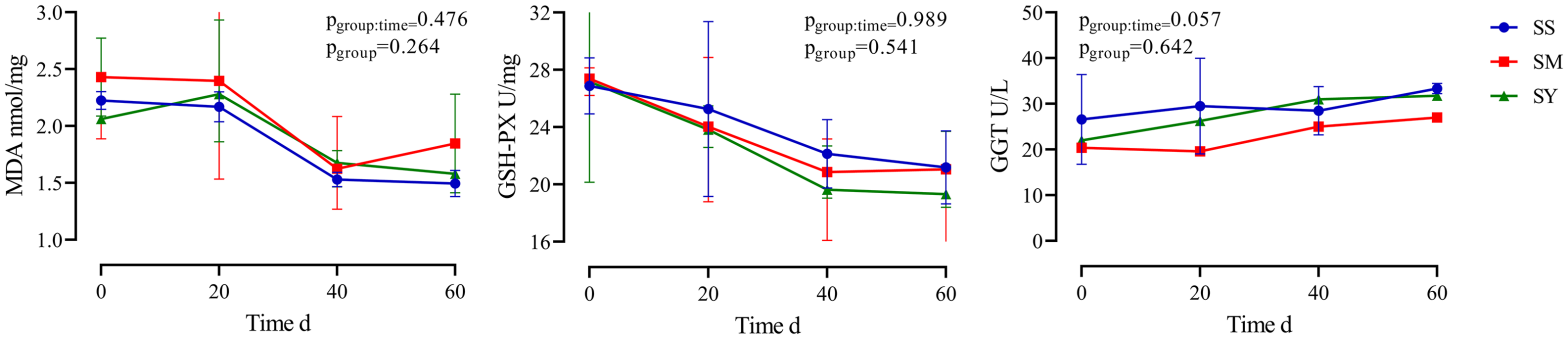
Effects of different Se sources on MDA concentrations and activities of GSH–Px and GGT in plasma of beef cattle (*n* = 5) over the 60 d feeding period. **(A)** malondialdehyde (MDA) concentrations; **(B)** antioxidant enzyme glutathione peroxidase (GSH-PX) activity; **(C)** gamma-glutamyl transpeptidase (GGT) activity. *p < 0.05, **p < 0.01. SS, diet supplemented 0.1 mg Se/kg DM from sodium selenite; SM, diet supplemented 0.1 mg Se/kg DM from selenomethionine; SY, diet supplemented 0.1 mg Se/kg DM from Se-enriched yeast.

The MDA content of the longissimus dorsi was lower (*p* < 0.05, Figure 4A) in the organic Se (SM and SY) groups than in the SS group. Supplementation with the different Se sources did not cause differences (*p* > 0.05) in the activity of GSH-PX in the longissimus dorsi, semimembranosus, semitendinosus, and liver of beef cattle (Figure 4B). Similarly, the level of GSH also did not vary (*p* > 0.05) in the longissimus dorsi and liver among the different Se source groups (Figure 4C). There were no differences (*p* > 0.05) in the plasma concentrations of GSH-PX, GGT and MDA on day 20, 40 and 60 among all the Se-supplemented groups (Figure 5).

## 4. Discussion

Se is an essential cofactor for deiodinases that are responsible for the activation and deactivation of thyroid hormone, and as such, Se supplementation often improves the growth performance of beef cattle fed a Se-deficient diet (26). Supplementation with organic and inorganic Se improved the growth rate in lambs, with organic Se being more effective than inorganic Se (27). Similar results have been reported for beef cattle and calves (28–30). However, in our study, organic Se did not affect the growth performance of beef cattle compared with inorganic Se. In agreement with our findings, Cozzi et al. observed no differences in the growth performance and slaughter traits of Charolais calves supplemented with different sources of inorganic and organic Se during the finishing period (31). The results of the study on the effects of different Se sources on the performance of beef cattle were inconclusive. Therefore, more research needs to be done. Se deposition in tissues and organs of cattle is selective, and most Se is preferentially deposited in the kidney, as it is the main site for excretion of Se from the body (19). Bitao et al. reported that organic Se sources were more effective in increasing kidney weight than inorganic Se source (32). Similarly, our study showed that beef cattle fed organic Se had a higher kidney weight and index than cattle fed inorganic Se. These results might be related to the preferential deposition of Se in the kidney, but this conjecture remains to be further studied.

**Figure 6:**
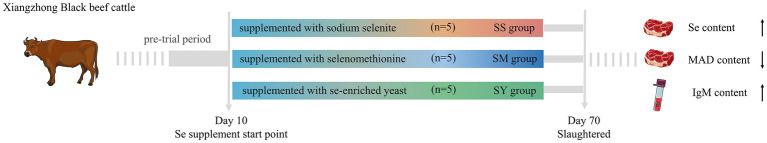
The Graphical abstract of this experiment.

Post-slaughtering deterioration of meat quality is predominantly caused by lipid oxidation. The extent of lipid oxidation during the shelf life affects meat quality characteristics such as color, flavor, texture, and nutritional value. Se plays a vital role in a large number of biological functions in animals, such as antioxidant activity. MDA is one of the metabolites of lipid peroxides and is usually used to evaluate the degree of peroxidation and cell damage in the body. Several studies have shown that dietary Se supplementation can improve meat quality, softness, and odor and reduce cholesterol deposition, postslaughter lipids oxidation and water loss (33, 34). It was reported that the effects of organic Se are better than those of inorganic Se in improving the serum and muscle antioxidant status of lambs and prolonging the muscle shelf life (35). Our results also showed that beef cattle fed organic Se had an improvement in the antioxidant status of the meat, as reflected by the significant reduction in MDA content compared to cattle fed inorganic Se, but no differences in meat quality were observed among the different Se sources. In agreement with our findings, Juniper et al. found that GSH-Px activity was significantly increased by organic Se sources compared to inorganic Se, but meat quality and stability were not affected (36). Silva et al. found that both organic and inorganic Se supplementation had no significant effect on carcass pH, drip loss, and cooking loss (37). And several authors also found no significant differences in the activity of GSH-PX in beef cattle (36), lamb (38), and calves (39) supplemented with different Se sources. In contrast, Cozzi et al. reported that organic Se has better meat quality than inorganic Se (31). Recently, Sun et al. reported that SM and hydroxy-selenomethionine (HMSeBA) increased the activity and mRNA abundance of GSH-Px and decreased the activities of SOD and ROS in bovine mammary epithelial cells compared with SS (40). Gong et al. suggested that dietary organic Se supplementation was more efficient in increasing GSH-PX activity and total antioxidant capacity, as well as decreasing the MDA content compared to inorganic Se supplementation (41). This discrepancy among the studies can be attributed to the amount of Se present in the basal diet, amount of supplemental Se, source of Se, and method of administration (19). In the study of Mehdi et al., Se was supplemented at 173 mg/kg DM to the diet of Belgian blue bulls, which significantly altered the chemical composition of meat and had no significant effect on meat quality (42). In our study, 0.1 mg Se/kg DM supplementation may not be enough to cause changes in meat quality and chemical composition. Further systematic and large-scale studies are required to assess the effects of the source and dosage of Se supplementation on the meat quality and antioxidant status of beef cattle.

Beef is one of the most important sources of dietary Se for human beings. Se-enriched beef and mutton products are those that contain more than 150 μg Se/kg. Cozzi et al. and Silva et al. reported that long-term supplementation of SY had higher Se concentrations in Nellore cattle muscle tissues than SS supplementation (31, 37). Similar results were obtained in pigs (43), lambs (44), chickens (45) and veals (46). Previous research suggests that organic Se can be more effectively stored in various tissues than inorganic Se (47). Rumen microbes decrease the bioavailability of inorganic Se, resulting in lower supply of Se to the body as compared to organic source (17). However, in the current study, the Se contents of muscles, organs and hair were not significantly different among the Se source groups. In addition, the Se content of muscle in different Se source groups was above 0.50 mg/kg, far exceeding the recommended level (150 μg Se/kg) of Se-enriched foods. Hintze et al. indicated that the initial Se content in the muscle of beef cattle in Se-deficient areas was 0.35 mg/kg, but the increase in Se content in muscle was not obvious (0.43 mg/kg) after dietary supplementation with 0.62 mg/kg Se (48). Silvia et al. added 0.2 mg/kg Se to the diet, and the highest Se content in muscle was 0.43 mg/kg (7). However, compared with these studies, a lower dietary Se supplementation level (0.1 mg/kg) resulted in a higher muscle Se content (0.50 mg/kg) in our study, which might be related to animal breed or indicate that Xiangzhong black beef cattle might have a better Se deposition ability. However, more research is needed to confirm these suspicions.

Plasma Se increases after dietary Se supplementation (19), and similar results were also observed in the current study. Some studies have shown that plasma Se concentrations are not affected by different Se sources (49, 50). However, the plasma Se concentration of the SM group on day 60 was lower than that of the SS and SY groups. The reason for this is not clear. Immunoglobulins, including IgG, IgA, and IgM, are proteins produced by plasma cells and form the key components of humoral immunity. The stimulating role of Se in immune function, such as T-cell proliferation and NK cell activation, was confirmed previously (51). Adequate amounts of Se in the body can promote the synthesis of antibodies and immunoglobulins, thereby improving the immune function of animals. A linear increase in plasma IgM concentration was observed in pregnant ewes in response to increasing dietary Se levels, and SY and SS sources had a similar impact (52). Our results showed that an organic Se source resulted in a higher plasma IgM concentration than an inorganic source. Gong et al. reported that organic Se supplementation significantly increased plasma IgA concentrations after 30 days, however, the concentrations of other immunoglobulins were not altered (41). Overall, these results showed that Se supplementation can improve immune function and that organic sources of Se are more effective than inorganic sources. This might be explained by organic Se having a greater ability to enhance liver and muscle selenoprotein gene expression than inorganic Se (53). In addition, organic Se is more efficient in inducing lymphocytes to secrete cytokines that are critical for humoral immunity initiation and immunoglobulin production (54).

It should be noted that the results of this study, especially the results of meat samples, come from a small sample size. However, the sensitive power analysis of (GPower 3.0.10) shows that the data in the present study are reliable. In spite of this, considering that a small sample size might increase the likelihood of type II errors, it is hoped that researchers will pay attention to this situation when referring to the results of this study.

## 5. Conclusion

Our study demonstrated that organic and inorganic sources of Se have similar effects on the growth performance, carcass characteristics, and meat quality of beef cattle. However, compared to inorganic Se sources, organic Se sources caused a significantly higher increase in plasma immunoglobulin and a decrease in MDA concentration in meat, demonstrating better bioavailability and antioxidant capacity of organic Se in Chinese Xiangzhong black beef cattle (Figure 6). Therefore, when considering the advantages of organic Se in improving immunity and antioxidant ability, selenomethionine (SM) or Se-enriched yeast (SY) can be a better choice for dietary Se supplementation.

## Data availability statement

The raw data supporting the conclusions of this article will be made available by the authors, without undue reservation.

## Ethics statement

The animal study was reviewed and approved by the animal care committee of the Institute of Subtropical Agriculture, Chinese Academy of Sciences, Changsha, China. Written informed consent was obtained from the owners for the participation of their animals in this study.

## Author contributions

XH conceived the project and designed the experiment. QH performed the experiment and collected and analysed samples. QH and XY performed the data analysis and drafted the manuscript. SW, YL, and NK performed the data curation and visualization, revised the manuscript, XH and ZT performed the funding acquisition, project administration, data curation, writing-review and editing and conceptualization, and supervision of the study. All authors contributed to the article and approved the submitted version.

## Funding

This work was supported by the Strategic Priority Research Program of the Chinese Academy of Sciences (Grant Nos. XDA26040304 and XDA26010301).

## Conflict of interest

The authors declare that the research was conducted in the absence of any commercial or financial relationships that could be construed as a potential conflict of interest.

## Publisher’s note

All claims expressed in this article are solely those of the authors and do not necessarily represent those of their affiliated organizations, or those of the publisher, the editors and the reviewers. Any product that may be evaluated in this article, or claim that may be made by its manufacturer, is not guaranteed or endorsed by the publisher.

## Figures and Tables

**Table:** 

ADF	acid detergent fibre
ADG	average daily gain
ALB	albumin
ALP	alkaline phosphatase
ALT	alanine aminotransferase
AST	aspartate transaminase
BW	body weight
CK	creatine kinase
CP	crude protein
CTAB	cetyltrimethylammonium bromide
DM	dry matter
EE	ether extract
FBW	final body weight
GGT	gamma-glutamyl transpeptidase
GSH	glutathione
GSH-PX	glutathione peroxidase
HCW	hot carcass weight
IBW	initial body weight
IgM	immunoglobulin M
MDA	malondialdehyde
NDF	neutral detergent fibre
NH_3_	ammonia
OM	organic matter
TP	total protein.
